# Poly[diethyl­enetriammonium [aquadi-μ_2_-sulfato-sulfatocerium(III)]]

**DOI:** 10.1107/S1600536810016600

**Published:** 2010-05-15

**Authors:** Xiu-Mei Zhang, Ya-Feng Li

**Affiliations:** a Department of X-ray, First Hospital, Jilin University, Changchun 130021, People’s Republic of China; b School of Chemical Engineering, Changchun University of Technology, Changchun 130012, People’s Republic of China

## Abstract

A new organically templated open-framework cerium sulfate, {(C_4_H_16_N_3_)[Ce(SO_4_)_3_(H_2_O)]}_*n*_, was hydro­thermally synthesized. The Ce^III^ cation is nine-coordinated by nine O atoms, including one water mol­ecule. Two of the SO_4_ groups afford one monodentate and bidentate linkages as the bridge to connect adjacent Ce^III^ cations, while the third SO_4_ group attaches the Ce^III^ cation in a bidentate mode. The crystal structure consists of layers composed of eight-membered-ring networks formed by four CeO_9_ polyhedra and four SO_4_ tetra­hedra. The triply protonated diethyl­enetriamine cations are located between adjacent layers and connect the layers *via* hydrogen bonds.

## Related literature

For related literature, see: Choudhury *et al.* (2001 ▶); Fu *et al.* (2006 ▶); Paul *et al.* (2002 ▶); Rao *et al.* (2006 ▶); Wickleder (2002 ▶).
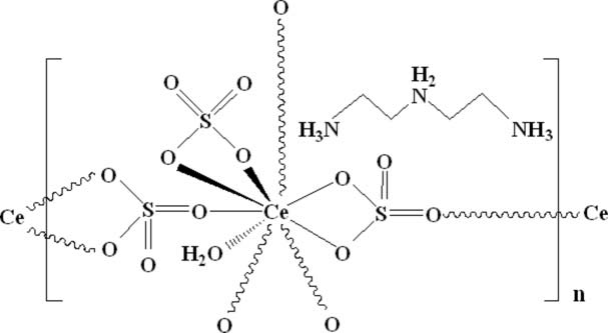


## Experimental

### 

#### Crystal data

(C_4_H_16_N_3_)[Ce(SO_4_)_3_(H_2_O)]
*M* *_r_* = 552.51
Monoclinic, 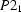

*a* = 6.6774 (13) Å
*b* = 10.397 (2) Å
*c* = 11.093 (2) Å
β = 93.77 (3)°
*V* = 768.5 (3) Å^3^
*Z* = 2
Mo *K*α radiation
μ = 3.44 mm^−1^
*T* = 293 K
0.25 × 0.22 × 0.19 mm

#### Data collection

Rigaku R-AXIS RAPID diffractometer
Absorption correction: empirical (using intensity measurements) (*ABSCOR*; Higashi, 1995 ▶) *T*_min_ = 0.480, *T*_max_ = 0.561
7575 measured reflections
3485 independent reflections
3443 reflections with *I* > 2σ(*I*)
*R*_int_ = 0.017

#### Refinement

*R*[*F* ^2^ > 2σ(*F* ^2^)] = 0.015
*wR*(*F* ^2^) = 0.041
*S* = 1.15
3485 reflections
225 parameters
4 restraints
H atoms treated by a mixture of independent and constrained refinement
Δρ_max_ = 0.56 e Å^−3^
Δρ_min_ = −0.71 e Å^−3^
Absolute structure: Flack (1983 ▶)
Flack parameter: −0.009 (8)

### 

Data collection: *PROCESS-AUTO* (Rigaku, 1998 ▶); cell refinement: *PROCESS-AUTO*; data reduction: *CrystalStructure* (Rigaku/MSC, 2002 ▶); program(s) used to solve structure: *SHELXS97* (Sheldrick, 2008 ▶); program(s) used to refine structure: *SHELXL97* (Sheldrick, 2008 ▶); molecular graphics: *DIAMOND* (Brandenburg, 2000 ▶); software used to prepare material for publication: *SHELXL97*.

## Supplementary Material

Crystal structure: contains datablocks I, global. DOI: 10.1107/S1600536810016600/hb5433sup1.cif

Structure factors: contains datablocks I. DOI: 10.1107/S1600536810016600/hb5433Isup2.hkl

Additional supplementary materials:  crystallographic information; 3D view; checkCIF report

## Figures and Tables

**Table 1:** Hydrogen-bond geometry (Å, °)

*D*—H⋯*A*	*D*—H	H⋯*A*	*D*⋯*A*	*D*—H⋯*A*
O1*W*—H1*F*⋯O4	0.83 (2)	1.98 (2)	2.766 (3)	159 (4)
O1*W*—H1*G*⋯O11^i^	0.81 (2)	2.06 (2)	2.850 (3)	164 (4)
N1—H1*A*⋯O8^ii^	0.89	2.02	2.769 (3)	141
N1—H1*C*⋯O9^ii^	0.89	2.02	2.883 (3)	162
N1—H1*B*⋯O6^iii^	0.89	2.05	2.852 (3)	150
N2—H2*B*⋯O11	0.90	1.92	2.764 (4)	156
N2—H2*A*⋯O2^iv^	0.90	2.16	2.993 (3)	154
N2—H2*A*⋯O4^iv^	0.90	2.30	2.997 (3)	134
N3—H3*A*⋯O5^v^	0.89	2.17	2.808 (3)	128
N3—H3*A*⋯O3^vi^	0.89	2.26	3.059 (4)	150
N3—H3*C*⋯O12^v^	0.89	1.91	2.799 (4)	173
N3—H3*B*⋯O10^vii^	0.89	2.04	2.763 (4)	137

Symmetry codes: (i) 
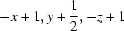
; (ii) 
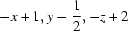
; (iii) 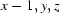; (iv) 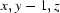; (v) 
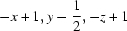
; (vi) 
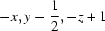
; (vii) 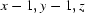.

**Fig. 1.**
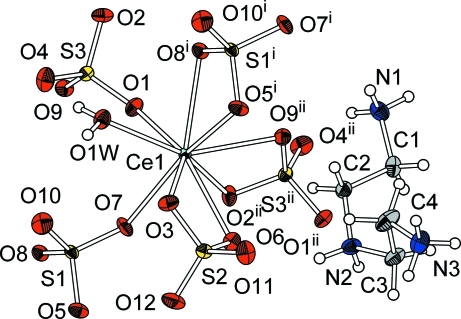
The unit cell of (I), showing the atomic labelling scheme and displacement ellipsoids at the 50% probability level. [Symmetry codes: (i) 1+x, y, z; (ii) 1-x, 0.5+y, -z.]

**Fig. 2.**
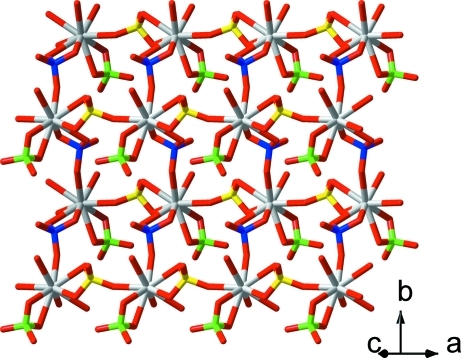
The stick plot of (I), displaying the layer along (101) direction composed by linking the Ce cation with µ2-S(1)O4 and µ2-S(3)O4. S(1) is shown in yellow, S(2) in green and S(3) in blue.

## supplementary crystallographic information

### Comment

The hydrous and anhydrous lanthanide sulfates have been intensively studied due to use of the separation of rare earth elements (Wickleder, 2002). Since the pioneering works of Rao *et al.* (Choudhury, *et al.*, 2001; Paul, *et al.*, 2002; Rao, *et al.*, 2006) on the preparation of organically templated open-framework metal sulfates, a remarkable plenty of organically templated open-framework rare-earth sulfates have been describled also. The example of organically templated cerium sulfate is few reported except for (C_4_H_12_N_2_)_4_^.^[Ce_8_(SO_4_)_16_(H_2_O)_8_] and (C_2_H_10_N_2_)_2_^.^[Ce_2_(SO_4_)_5_(H_2_O)_2_] (Fu, *et al.*, 2006). In this work, a new layer cerium sulfate, {(C_4_H_16_N_3_)[Ce(SO_4_)_3_(H_2_O)]}_n_, is obtained.

The asymmetric unit of (I) comprises of one Ce^III^ cation, three SO_4_ groups, one coordination water and one protonated diethylene triamine cation, as shown in Fig.1. The Ce^III^ cation is 9-coordinated by nine oxygen including one water molecule with the bond distances from 2.468 (2) Å to 2.588 (27) Å and the angles of O—Ce—O between 54.18 (10)° and 149.13 (10)°. Three SO_4_ can be divided into two modes: S(1) and S(3) consist of three S—O—Ce linkages and links adjacent Ce atoms through one bidentate and one monodentate; S(2) makes two S—O—Ce linkages as a ligand of one Ce atom through bidentate. The bond angles of S—O—Ce of bidentate coordination range from 99.23 (10)° to 101.8 (1)°, and the S—O—Ce of monodentate coordination is at 141.81 (9)° and 144.17 (13)°.

As shown in Fig.2, the layer of (I) is accomplished by connect the Ce cations by µ_2_-S(1)O_4_ and µ_2_-S(3)O_4_ as the bridge along (100) and (010) direction, respectively. The S(2)O_4_ do not take part in the formation of layer and coordinates to Ce cation by the bidentate mode. The protonated H_3_DETA interacts with the layer by the H-bond of N—H···O.

### Experimental

(I) was synthesized under hydrothermal condition. In a typically route, Ce(NO_3_)_3_^.^6H_2_O (0.30 g, 0.7 mmol) was dissolved in 5 ml deionized water under stirring, and then H_2_SO_4_ (95%, 0.25 ml, 4.55 mmol) and DETA (0.22 ml, 2.8 mmol) were dropwisely added to a clear solution with pH=3.0. After continuously stirred for 3 h, the solution with the molar ratio of Ce(NO_3_)_3_^.^6H_2_O : 6.5H_2_SO_4_ : 2.8DETA : 397H_2_O was transferred into 23 ml autoclave and heated at 438 K for 5 days. After naturally cooling to room temperature, colorless block soluble product was collected by filtration as a single phase. The atomic ratio of Ce : S determined by EDX was 1 : 3, in consistence with the results of structural determination of (I).

### Refinement

Water H atoms were located in a difference Fourier map and were refined with O—H = 0.82 (2) Å, H···H = 1.37 (2) Å and Uiso(H) = 1.2Ueq(O). The remaining H-atoms were placed in calculated positions (C—H = 0.89 Å, N—H = 0.89-0.90 Å) and were included in the refinement in the riding-model approximation, with U(H) = 1.2Ueq(C, N).

### Crystal data

**Table:**

(C_4_H_16_N_3_)[Ce(SO_4_)_3_(H_2_O)]	*F*(000) = 546
*M**_r_* = 552.51	*D*_x_ = 2.388 Mg m^−^^3^
Monoclinic, *P*2_1_	Mo *K*α radiation, λ = 0.71073 Å
Hall symbol: P 2yb	Cell parameters from 1000 reflections
*a* = 6.6774 (13) Å	θ = 3.1–24.8°
*b* = 10.397 (2) Å	µ = 3.44 mm^−^^1^
*c* = 11.093 (2) Å	*T* = 293 K
β = 93.77 (3)°	Block, colorless
*V* = 768.5 (3) Å^3^	0.25 × 0.22 × 0.19 mm
*Z* = 2	

### Data collection

**Table:**

Rigaku R-AXIS RAPID diffractometer	3485 independent reflections
Radiation source: fine-focus sealed tube	3443 reflections with *I* > 2σ(*I*)
graphite	*R*_int_ = 0.017
Detector resolution: 10.00 pixels mm^-1^	θ_max_ = 27.5°, θ_min_ = 3.1°
ω scans	*h* = −7→8
Absorption correction: empirical (using intensity measurements) (*ABSCOR*; Higashi, 1995)	*k* = −13→13
*T*_min_ = 0.480, *T*_max_ = 0.561	*l* = −14→14
7575 measured reflections	

### Refinement

**Table:**

Refinement on *F*^2^	Secondary atom site location: difference Fourier map
Least-squares matrix: full	Hydrogen site location: inferred from neighbouring sites
*R*[*F*^2^ > 2σ(*F*^2^)] = 0.015	H atoms treated by a mixture of independent and constrained refinement
*wR*(*F*^2^) = 0.041	*w* = 1/[σ^2^(*F*_o_^2^) + (0.005*P*)^2^ + 0.1027*P*] where *P* = (*F*_o_^2^ + 2*F*_c_^2^)/3
*S* = 1.15	(Δ/σ)_max_ = 0.001
3485 reflections	Δρ_max_ = 0.56 e Å^−^^3^
225 parameters	Δρ_min_ = −0.71 e Å^−^^3^
4 restraints	Absolute structure: Flack (1983)
Primary atom site location: structure-invariant direct methods	Flack parameter: −0.009 (8)

### Special details

**Table:**

Geometry. All esds (except the esd in the dihedral angle between two l.s. planes) are estimated using the full covariance matrix. The cell esds are taken into account individually in the estimation of esds in distances, angles and torsion angles; correlations between esds in cell parameters are only used when they are defined by crystal symmetry. An approximate (isotropic) treatment of cell esds is used for estimating esds involving l.s. planes.
Refinement. Refinement of *F*^2^ against ALL reflections. The weighted *R*-factor wR and goodness of fit *S* are based on *F*^2^, conventional *R*-factors *R* are based on *F*, with *F* set to zero for negative *F*^2^. The threshold expression of *F*^2^ > σ(*F*^2^) is used only for calculating *R*-factors(gt) etc. and is not relevant to the choice of reflections for refinement. *R*-factors based on *F*^2^ are statistically about twice as large as those based on *F*, and *R*- factors based on ALL data will be even larger.

### Fractional atomic coordinates and isotropic or equivalent isotropic displacement parameters (Å^2^)

**Table:**

	*x*	*y*	*z*	*U*_iso_*/*U*_eq_	
Ce1	0.468643 (16)	0.636197 (16)	0.819119 (10)	0.00928 (4)	
S1	1.00549 (10)	0.68984 (7)	0.75724 (6)	0.01204 (13)	
S2	0.57509 (11)	0.41208 (7)	0.63748 (6)	0.01388 (14)	
S3	0.57345 (10)	0.97128 (6)	0.93682 (6)	0.01258 (13)	
O1	0.5483 (3)	0.8315 (2)	0.9420 (2)	0.0190 (4)	
O2	0.3896 (3)	1.0370 (2)	0.97444 (19)	0.0198 (4)	
O3	0.5087 (3)	0.5445 (2)	0.60536 (19)	0.0199 (4)	
O4	0.6164 (4)	1.0119 (2)	0.81523 (19)	0.0249 (5)	
O5	1.1298 (3)	0.5804 (2)	0.71992 (18)	0.0181 (4)	
O6	0.5651 (3)	0.40504 (19)	0.77192 (18)	0.0172 (4)	
O7	0.8387 (2)	0.6423 (3)	0.82503 (16)	0.0198 (4)	
O8	1.1447 (3)	0.7667 (2)	0.83878 (18)	0.0160 (4)	
O9	0.7341 (3)	1.01219 (19)	1.02740 (19)	0.0178 (4)	
O10	0.9302 (3)	0.7643 (2)	0.6533 (2)	0.0264 (5)	
O11	0.4326 (3)	0.3179 (2)	0.5804 (2)	0.0242 (5)	
O12	0.7762 (3)	0.3875 (2)	0.6036 (2)	0.0276 (5)	
O1W	0.4833 (4)	0.8105 (2)	0.66809 (19)	0.0199 (4)	
H1F	0.529 (5)	0.879 (2)	0.696 (3)	0.024*	
H1G	0.520 (5)	0.799 (3)	0.600 (2)	0.024*	
N1	−0.0899 (4)	0.3572 (3)	0.9309 (2)	0.0214 (5)	
H1A	−0.1665	0.3382	0.9912	0.026*	
H1B	−0.1653	0.3921	0.8703	0.026*	
H1C	0.0049	0.4127	0.9567	0.026*	
N2	0.2464 (4)	0.1635 (2)	0.7422 (2)	0.0204 (6)	
H2A	0.3274	0.1334	0.8039	0.025*	
H2B	0.3257	0.1928	0.6856	0.025*	
N3	−0.0952 (4)	−0.0122 (3)	0.5183 (2)	0.0245 (6)	
H3A	−0.1851	0.0142	0.4609	0.029*	
H3B	−0.1518	−0.0693	0.5653	0.029*	
H3C	0.0079	−0.0486	0.4845	0.029*	
C1	0.0052 (5)	0.2382 (3)	0.8884 (3)	0.0223 (6)	
H1D	−0.0969	0.1769	0.8604	0.027*	
H1E	0.0873	0.1992	0.9540	0.027*	
C2	0.1330 (5)	0.2739 (3)	0.7870 (3)	0.0182 (6)	
H2C	0.0477	0.3090	0.7208	0.022*	
H2D	0.2268	0.3405	0.8147	0.022*	
C3	0.1272 (5)	0.0532 (3)	0.6894 (3)	0.0234 (7)	
H3D	0.0579	0.0108	0.7524	0.028*	
H3E	0.2169	−0.0087	0.6558	0.028*	
C4	−0.0234 (5)	0.0989 (3)	0.5919 (3)	0.0279 (8)	
H4A	−0.1355	0.1398	0.6280	0.034*	
H4B	0.0383	0.1615	0.5412	0.034*	

### Atomic displacement parameters (Å^2^)

**Table:**

	*U*^11^	*U*^22^	*U*^33^	*U*^12^	*U*^13^	*U*^23^
Ce1	0.00800 (7)	0.00962 (6)	0.01010 (6)	−0.00022 (6)	−0.00020 (5)	−0.00028 (7)
S1	0.0082 (3)	0.0151 (3)	0.0127 (3)	−0.0003 (2)	−0.0003 (3)	0.0010 (2)
S2	0.0137 (3)	0.0150 (3)	0.0128 (3)	0.0005 (3)	−0.0002 (3)	−0.0029 (2)
S3	0.0141 (3)	0.0122 (3)	0.0113 (3)	−0.0004 (2)	−0.0007 (3)	−0.0016 (2)
O1	0.0245 (11)	0.0130 (10)	0.0190 (10)	−0.0016 (8)	−0.0016 (9)	−0.0026 (8)
O2	0.0165 (10)	0.0207 (10)	0.0216 (10)	0.0066 (8)	−0.0027 (9)	−0.0042 (8)
O3	0.0222 (11)	0.0194 (10)	0.0178 (10)	0.0059 (8)	−0.0012 (9)	0.0004 (8)
O4	0.0369 (13)	0.0251 (12)	0.0131 (10)	−0.0070 (10)	0.0043 (10)	0.0000 (8)
O5	0.0118 (9)	0.0214 (10)	0.0208 (10)	0.0010 (8)	−0.0019 (9)	−0.0082 (8)
O6	0.0212 (10)	0.0181 (10)	0.0118 (9)	−0.0005 (8)	−0.0022 (9)	0.0000 (8)
O7	0.0102 (7)	0.0279 (10)	0.0216 (8)	−0.0029 (11)	0.0029 (7)	0.0037 (12)
O8	0.0133 (9)	0.0177 (10)	0.0171 (9)	−0.0015 (7)	0.0012 (8)	−0.0040 (8)
O9	0.0142 (9)	0.0185 (10)	0.0202 (10)	−0.0012 (8)	−0.0033 (9)	−0.0053 (8)
O10	0.0243 (11)	0.0317 (13)	0.0220 (11)	0.0019 (10)	−0.0074 (10)	0.0097 (9)
O11	0.0277 (12)	0.0270 (12)	0.0176 (10)	−0.0095 (10)	−0.0012 (10)	−0.0062 (9)
O12	0.0199 (10)	0.0321 (13)	0.0317 (12)	0.0067 (9)	0.0077 (10)	−0.0061 (10)
O1W	0.0268 (11)	0.0179 (11)	0.0153 (10)	−0.0013 (10)	0.0034 (9)	0.0011 (8)
N1	0.0179 (13)	0.0301 (15)	0.0164 (11)	−0.0004 (10)	0.0017 (11)	0.0016 (10)
N2	0.0166 (11)	0.0171 (16)	0.0268 (12)	0.0019 (9)	−0.0046 (11)	−0.0027 (9)
N3	0.0334 (15)	0.0221 (14)	0.0174 (12)	−0.0037 (11)	−0.0025 (12)	0.0021 (10)
C1	0.0245 (16)	0.0212 (15)	0.0213 (14)	−0.0046 (12)	0.0027 (13)	0.0043 (11)
C2	0.0202 (14)	0.0151 (14)	0.0193 (13)	−0.0008 (11)	0.0025 (12)	−0.0001 (11)
C3	0.0255 (16)	0.0140 (14)	0.0300 (16)	0.0022 (11)	−0.0046 (14)	−0.0006 (12)
C4	0.0401 (19)	0.0169 (15)	0.0250 (15)	0.0030 (12)	−0.0111 (16)	−0.0025 (11)

### Geometric parameters (Å, °)

**Table:**

Ce1—O7	2.4685 (17)	N1—C1	1.481 (4)
Ce1—O1W	2.474 (2)	N1—H1A	0.8900
Ce1—O1	2.484 (2)	N1—H1B	0.8900
Ce1—O5^i^	2.518 (2)	N1—H1C	0.8900
Ce1—O6	2.551 (2)	N2—C2	1.479 (4)
Ce1—O8^i^	2.575 (2)	N2—C3	1.493 (4)
Ce1—O3	2.586 (2)	N2—H2A	0.9000
Ce1—O9^ii^	2.588 (2)	N2—H2B	0.9000
Ce1—O2^ii^	2.631 (2)	N3—C4	1.476 (4)
S1—O10	1.451 (2)	N3—H3A	0.8900
S1—O7	1.470 (2)	N3—H3B	0.8900
S1—O5	1.483 (2)	N3—H3C	0.8900
S1—O8	1.487 (2)	C1—C2	1.502 (4)
S2—O12	1.441 (2)	C1—H1D	0.9700
S2—O11	1.478 (2)	C1—H1E	0.9700
S2—O3	1.482 (2)	C2—H2C	0.9700
S2—O6	1.499 (2)	C2—H2D	0.9700
S3—O4	1.460 (2)	C3—C4	1.504 (4)
S3—O1	1.465 (2)	C3—H3D	0.9700
S3—O9	1.483 (2)	C3—H3E	0.9700
S3—O2	1.488 (2)	C4—H4A	0.9700
O1W—H1F	0.828 (18)	C4—H4B	0.9700
O1W—H1G	0.813 (18)		
			
O7—Ce1—O1W	85.13 (8)	O11—S2—O3	109.76 (14)
O7—Ce1—O1	77.68 (8)	O11—S2—O3	109.76 (14)
O1W—Ce1—O1	75.90 (8)	O12—S2—O6	110.75 (13)
O7—Ce1—O5^i^	152.47 (7)	O11—S2—O6	108.94 (13)
O1W—Ce1—O5^i^	86.97 (8)	O11—S2—O6	108.94 (13)
O1—Ce1—O5^i^	125.59 (7)	O3—S2—O6	104.62 (12)
O7—Ce1—O6	76.34 (8)	O12—S2—Ce1	122.98 (10)
O1W—Ce1—O6	121.95 (7)	O11—S2—Ce1	126.18 (10)
O1—Ce1—O6	146.61 (7)	O11—S2—Ce1	126.18 (10)
O5^i^—Ce1—O6	85.70 (7)	O3—S2—Ce1	53.00 (8)
O7—Ce1—O8^i^	146.03 (8)	O6—S2—Ce1	51.77 (8)
O1W—Ce1—O8^i^	75.06 (7)	O4—S3—O1	110.70 (14)
O1—Ce1—O8^i^	70.94 (7)	O4—S3—O1	110.70 (14)
O5^i^—Ce1—O8^i^	54.73 (6)	O4—S3—O9	111.45 (13)
O6—Ce1—O8^i^	137.61 (6)	O4—S3—O9	111.45 (13)
O7—Ce1—O3	82.53 (7)	O1—S3—O9	109.74 (12)
O1W—Ce1—O3	68.78 (7)	O4—S3—O2	109.99 (14)
O1—Ce1—O3	140.66 (7)	O4—S3—O2	109.99 (14)
O5^i^—Ce1—O3	70.03 (7)	O1—S3—O2	110.21 (14)
O6—Ce1—O3	54.67 (6)	O9—S3—O2	104.58 (12)
O8^i^—Ce1—O3	114.22 (7)	O4—S3—Ce1^iv^	130.80 (10)
O7—Ce1—O9^ii^	124.15 (7)	O4—S3—Ce1^iv^	130.80 (10)
O1W—Ce1—O9^ii^	148.88 (7)	O1—S3—Ce1^iv^	118.49 (10)
O1—Ce1—O9^ii^	98.55 (7)	O9—S3—Ce1^iv^	51.63 (9)
O5^i^—Ce1—O9^ii^	71.27 (7)	O2—S3—Ce1^iv^	53.35 (8)
O6—Ce1—O9^ii^	79.38 (7)	S3—O1—Ce1	144.17 (13)
O8^i^—Ce1—O9^ii^	74.23 (7)	S3—O2—Ce1^iv^	99.66 (10)
O3—Ce1—O9^ii^	120.64 (7)	S2—O3—Ce1	99.75 (10)
O7—Ce1—O2^ii^	71.62 (7)	S1—O5—Ce1^iii^	101.82 (10)
O1W—Ce1—O2^ii^	148.08 (7)	S2—O6—Ce1	100.75 (10)
O1—Ce1—O2^ii^	77.94 (7)	S1—O7—Ce1	141.80 (12)
O5^i^—Ce1—O2^ii^	123.49 (7)	S1—O8—Ce1^iii^	99.21 (10)
O6—Ce1—O2^ii^	74.22 (7)	S3—O9—Ce1^iv^	101.66 (10)
O8^i^—Ce1—O2^ii^	112.91 (7)	Ce1—O1W—H1F	114 (3)
O3—Ce1—O2^ii^	126.92 (7)	Ce1—O1W—H1G	123 (3)
O9^ii^—Ce1—O2^ii^	53.54 (6)	H1F—O1W—H1G	110 (3)
O7—Ce1—S1^i^	163.95 (6)	C1—N1—H1A	109.5
O1W—Ce1—S1^i^	78.82 (6)	C1—N1—H1B	109.5
O1—Ce1—S1^i^	98.37 (5)	H1A—N1—H1B	109.5
O5^i^—Ce1—S1^i^	27.23 (5)	C1—N1—H1C	109.5
O6—Ce1—S1^i^	112.13 (5)	H1A—N1—H1C	109.5
O8^i^—Ce1—S1^i^	27.55 (4)	H1B—N1—H1C	109.5
O3—Ce1—S1^i^	91.41 (6)	C2—N2—C3	117.1 (2)
O9^ii^—Ce1—S1^i^	71.66 (5)	C2—N2—H2A	108.0
O2^ii^—Ce1—S1^i^	123.17 (5)	C3—N2—H2A	108.0
O7—Ce1—S2	76.78 (6)	C2—N2—H2B	108.0
O1W—Ce1—S2	94.98 (6)	C3—N2—H2B	108.0
O1—Ce1—S2	153.51 (6)	H2A—N2—H2B	107.3
O5^i^—Ce1—S2	77.70 (5)	C4—N3—H3A	109.5
O6—Ce1—S2	27.48 (4)	C4—N3—H3B	109.5
O8^i^—Ce1—S2	131.42 (5)	H3A—N3—H3B	109.5
O3—Ce1—S2	27.25 (5)	C4—N3—H3C	109.5
O9^ii^—Ce1—S2	101.51 (5)	H3A—N3—H3C	109.5
O2^ii^—Ce1—S2	100.43 (5)	H3B—N3—H3C	109.5
S1^i^—Ce1—S2	104.25 (3)	N1—C1—C2	108.0 (2)
O7—Ce1—S3^ii^	97.66 (6)	N1—C1—H1D	110.1
O1W—Ce1—S3^ii^	164.83 (5)	C2—C1—H1D	110.1
O1—Ce1—S3^ii^	90.07 (5)	N1—C1—H1E	110.1
O5^i^—Ce1—S3^ii^	96.83 (6)	C2—C1—H1E	110.1
O6—Ce1—S3^ii^	73.10 (5)	H1D—C1—H1E	108.4
O8^i^—Ce1—S3^ii^	95.10 (5)	N2—C2—C1	112.9 (2)
O3—Ce1—S3^ii^	126.32 (5)	N2—C2—H2C	109.0
O9^ii^—Ce1—S3^ii^	26.70 (4)	C1—C2—H2C	109.0
O2^ii^—Ce1—S3^ii^	26.99 (4)	N2—C2—H2D	109.0
S1^i^—Ce1—S3^ii^	97.89 (3)	C1—C2—H2D	109.0
S2—Ce1—S3^ii^	100.18 (2)	H2C—C2—H2D	107.8
O10—S1—O7	110.58 (13)	N2—C3—C4	110.7 (2)
O10—S1—O5	111.01 (14)	N2—C3—H3D	109.5
O7—S1—O5	109.98 (15)	C4—C3—H3D	109.5
O10—S1—O8	111.51 (13)	N2—C3—H3E	109.5
O7—S1—O8	109.51 (12)	C4—C3—H3E	109.5
O5—S1—O8	104.08 (11)	H3D—C3—H3E	108.1
O10—S1—Ce1^iii^	123.20 (11)	N3—C4—C3	109.2 (3)
O7—S1—Ce1^iii^	126.20 (9)	N3—C4—H4A	109.8
O5—S1—Ce1^iii^	50.96 (8)	C3—C4—H4A	109.8
O8—S1—Ce1^iii^	53.24 (8)	N3—C4—H4B	109.8
O12—S2—O11	110.80 (15)	C3—C4—H4B	109.8
O12—S2—O11	110.80 (15)	H4A—C4—H4B	108.3
O12—S2—O3	111.77 (14)		
			
O7—Ce1—S2—O12	−5.76 (14)	O4—S3—O2—Ce1^iv^	−126.59 (11)
O1W—Ce1—S2—O12	78.04 (14)	O4—S3—O2—Ce1^iv^	−126.59 (11)
O1—Ce1—S2—O12	9.97 (17)	O1—S3—O2—Ce1^iv^	111.08 (11)
O5^i^—Ce1—S2—O12	163.83 (14)	O9—S3—O2—Ce1^iv^	−6.81 (12)
O6—Ce1—S2—O12	−91.51 (16)	O12—S2—O3—Ce1	−115.66 (13)
O8^i^—Ce1—S2—O12	152.48 (14)	O11—S2—O3—Ce1	120.98 (12)
O3—Ce1—S2—O12	93.70 (17)	O11—S2—O3—Ce1	120.98 (12)
O9^ii^—Ce1—S2—O12	−128.49 (13)	O6—S2—O3—Ce1	4.23 (12)
O2^ii^—Ce1—S2—O12	−73.88 (13)	O7—Ce1—O3—S2	75.58 (12)
S1^i^—Ce1—S2—O12	157.75 (12)	O1W—Ce1—O3—S2	163.23 (14)
S3^ii^—Ce1—S2—O12	−101.32 (13)	O1—Ce1—O3—S2	135.61 (11)
O7—Ce1—S2—O11	172.06 (13)	O5^i^—Ce1—O3—S2	−102.14 (12)
O1W—Ce1—S2—O11	−104.14 (14)	O6—Ce1—O3—S2	−2.95 (8)
O1—Ce1—S2—O11	−172.22 (17)	O8^i^—Ce1—O3—S2	−135.32 (10)
O5^i^—Ce1—S2—O11	−18.35 (13)	O9^ii^—Ce1—O3—S2	−49.90 (13)
O6—Ce1—S2—O11	86.31 (16)	O2^ii^—Ce1—O3—S2	15.33 (15)
O8^i^—Ce1—S2—O11	−29.70 (14)	S1^i^—Ce1—O3—S2	−119.34 (10)
O3—Ce1—S2—O11	−88.48 (17)	S3^ii^—Ce1—O3—S2	−18.47 (13)
O9^ii^—Ce1—S2—O11	49.33 (13)	O1—S3—O4—O4	0.0 (7)
O2^ii^—Ce1—S2—O11	103.93 (13)	O9—S3—O4—O4	0.0 (7)
S1^i^—Ce1—S2—O11	−24.43 (12)	O2—S3—O4—O4	0.0 (7)
S3^ii^—Ce1—S2—O11	76.49 (12)	Ce1^iv^—S3—O4—O4	0.0 (8)
O7—Ce1—S2—O11	172.06 (13)	O10—S1—O5—Ce1^iii^	116.35 (13)
O1W—Ce1—S2—O11	−104.14 (14)	O7—S1—O5—Ce1^iii^	−120.96 (10)
O1—Ce1—S2—O11	−172.22 (17)	O8—S1—O5—Ce1^iii^	−3.73 (13)
O5^i^—Ce1—S2—O11	−18.35 (13)	O12—S2—O6—Ce1	116.27 (13)
O6—Ce1—S2—O11	86.31 (16)	O11—S2—O6—Ce1	−121.62 (12)
O8^i^—Ce1—S2—O11	−29.70 (14)	O11—S2—O6—Ce1	−121.62 (12)
O3—Ce1—S2—O11	−88.48 (17)	O3—S2—O6—Ce1	−4.30 (12)
O9^ii^—Ce1—S2—O11	49.33 (13)	O7—Ce1—O6—S2	−87.55 (11)
O2^ii^—Ce1—S2—O11	103.93 (13)	O1W—Ce1—O6—S2	−12.30 (13)
S1^i^—Ce1—S2—O11	−24.43 (12)	O1—Ce1—O6—S2	−127.41 (12)
S3^ii^—Ce1—S2—O11	76.49 (12)	O5^i^—Ce1—O6—S2	71.42 (11)
O7—Ce1—S2—O3	−99.46 (13)	O8^i^—Ce1—O6—S2	91.15 (13)
O1W—Ce1—S2—O3	−15.66 (13)	O3—Ce1—O6—S2	2.92 (8)
O1—Ce1—S2—O3	−83.74 (16)	O9^ii^—Ce1—O6—S2	143.15 (11)
O5^i^—Ce1—S2—O3	70.13 (13)	O2^ii^—Ce1—O6—S2	−161.97 (11)
O6—Ce1—S2—O3	174.79 (15)	S1^i^—Ce1—O6—S2	78.11 (10)
O8^i^—Ce1—S2—O3	58.78 (13)	S3^ii^—Ce1—O6—S2	169.90 (10)
O9^ii^—Ce1—S2—O3	137.81 (12)	O10—S1—O7—Ce1	3.9 (3)
O2^ii^—Ce1—S2—O3	−167.59 (12)	O5—S1—O7—Ce1	−119.0 (2)
S1^i^—Ce1—S2—O3	64.05 (11)	O8—S1—O7—Ce1	127.2 (2)
S3^ii^—Ce1—S2—O3	164.97 (11)	Ce1^iii^—S1—O7—Ce1	−174.67 (17)
O7—Ce1—S2—O6	85.75 (12)	O1W—Ce1—O7—S1	−16.4 (3)
O1W—Ce1—S2—O6	169.55 (12)	O1—Ce1—O7—S1	−93.0 (3)
O1—Ce1—S2—O6	101.48 (15)	O5^i^—Ce1—O7—S1	57.5 (4)
O5^i^—Ce1—S2—O6	−104.66 (12)	O6—Ce1—O7—S1	108.2 (3)
O8^i^—Ce1—S2—O6	−116.01 (12)	O8^i^—Ce1—O7—S1	−70.3 (3)
O3—Ce1—S2—O6	−174.79 (15)	O3—Ce1—O7—S1	52.8 (3)
O9^ii^—Ce1—S2—O6	−36.98 (11)	O9^ii^—Ce1—O7—S1	175.0 (2)
O2^ii^—Ce1—S2—O6	17.63 (11)	O2^ii^—Ce1—O7—S1	−174.2 (3)
S1^i^—Ce1—S2—O6	−110.74 (10)	S1^i^—Ce1—O7—S1	−15.7 (5)
S3^ii^—Ce1—S2—O6	−9.81 (10)	S2—Ce1—O7—S1	79.9 (3)
O4—S3—O1—Ce1	−21.6 (3)	S3^ii^—Ce1—O7—S1	178.6 (3)
O4—S3—O1—Ce1	−21.6 (3)	O10—S1—O8—Ce1^iii^	−116.13 (12)
O9—S3—O1—Ce1	−145.0 (2)	O7—S1—O8—Ce1^iii^	121.17 (12)
O2—S3—O1—Ce1	100.3 (2)	O5—S1—O8—Ce1^iii^	3.62 (12)
Ce1^iv^—S3—O1—Ce1	158.70 (17)	O4—S3—O9—Ce1^iv^	125.76 (12)
O7—Ce1—O1—S3	93.1 (2)	O4—S3—O9—Ce1^iv^	125.76 (12)
O1W—Ce1—O1—S3	5.1 (2)	O1—S3—O9—Ce1^iv^	−111.24 (12)
O5^i^—Ce1—O1—S3	−70.6 (3)	O2—S3—O9—Ce1^iv^	6.96 (13)
O6—Ce1—O1—S3	132.7 (2)	O12—S2—O11—O11	0.0 (2)
O8^i^—Ce1—O1—S3	−73.7 (2)	O3—S2—O11—O11	0.0 (2)
O3—Ce1—O1—S3	31.6 (3)	O6—S2—O11—O11	0.00 (19)
O9^ii^—Ce1—O1—S3	−143.6 (2)	Ce1—S2—O11—O11	0.00 (19)
O2^ii^—Ce1—O1—S3	166.7 (2)	C3—N2—C2—C1	61.8 (3)
S1^i^—Ce1—O1—S3	−71.1 (2)	N1—C1—C2—N2	176.3 (2)
S2—Ce1—O1—S3	77.5 (3)	C2—N2—C3—C4	53.3 (4)
S3^ii^—Ce1—O1—S3	−169.0 (2)	N2—C3—C4—N3	163.5 (3)

Symmetry codes: (i) *x*−1, *y*, *z*; (ii) −*x*+1, *y*−1/2, −*z*+2; (iii) *x*+1, *y*, *z*;  (iv) −*x*+1, *y*+1/2, −*z*+2.

### Hydrogen-bond geometry (Å, °)

**Table:**

*D*—H···*A*	*D*—H	H···*A*	*D*···*A*	*D*—H···*A*
O1W—H1F···O4	0.83 (2)	1.98 (2)	2.766 (3)	159 (4)
O1W—H1G···O11^v^	0.81 (2)	2.06 (2)	2.850 (3)	164 (4)
N1—H1A···O8^ii^	0.89	2.02	2.769 (3)	141
N1—H1C···O9^ii^	0.89	2.02	2.883 (3)	162
N1—H1B···O6^i^	0.89	2.05	2.852 (3)	150
N2—H2B···O11	0.90	1.92	2.764 (4)	156
N2—H2A···O2^vi^	0.90	2.16	2.993 (3)	154
N2—H2A···O4^vi^	0.90	2.30	2.997 (3)	134
N3—H3A···O5^vii^	0.89	2.17	2.808 (3)	128
N3—H3A···O3^viii^	0.89	2.26	3.059 (4)	150
N3—H3C···O12^vii^	0.89	1.91	2.799 (4)	173
N3—H3B···O10^ix^	0.89	2.04	2.763 (4)	137

Symmetry codes:  (v) −*x*+1, *y*+1/2, −*z*+1; (ii) −*x*+1, *y*−1/2, −*z*+2; (i) *x*−1, *y*, *z*; (vi) *x*, *y*−1, *z*; (vii) −*x*+1, *y*−1/2, −*z*+1; (viii) −*x*, *y*−1/2, −*z*+1; (ix) *x*−1, *y*−1, *z*.

## References

[bb1] Brandenburg, K. (2000). *DIAMOND* Crystal Impact GbR, Bonn, Germany.

[bb2] Choudhury, A., Krishnamoorthy, J. & Rao, C. N. R. (2001). *Chem. Commun.* pp. 2610–2611.

[bb3] Flack, H. D. (1983). *Acta Cryst.* A**39**, 876–881.

[bb4] Fu, Y., Xu, Z. & Ren, J. (2006). *J. Mol. Struct.* **788**, 190–193.

[bb5] Higashi, T. (1995). *ABSCOR* Rigaku Corporation, Tokyo, Japan.

[bb6] Paul, G., Choudhury, A. & Rao, C. N. R. (2002). *J. Chem. Soc. Dalton Trans.* pp. 3859–3867.

[bb7] Rao, C. N. R., Behera, J. N. & Dan, M. (2006). *Chem. Soc. Rev.* **35**, 375–387. 10.1039/b510396g 16565754

[bb8] Rigaku (1998). *PROCESS-AUTO* Rigaku Corporation, Tokyo, Japan.

[bb9] Rigaku/MSC (2002). *CrystalStructure.* Rigaku/MSC, The Woodlands, Texas, USA.

[bb10] Sheldrick, G. M. (2008). *Acta Cryst.* A**64**, 112–122. 10.1107/S0108767307043930 18156677

[bb11] Wickleder, M. S. (2002). *Chem. Rev.* **102**, 2011–2087. 10.1021/cr010308o 12059261

